# UNAIDS 90-90-90—Opportunity in Every Difficulty

**DOI:** 10.1177/2325958218809075

**Published:** 2018-10-25

**Authors:** José M. Zuniga

**Affiliations:** 1 International Association of Providers of AIDS Care, Washington, DC, USA

The 22nd International AIDS Conference (AIDS 2018) in Amsterdam delivered a wake-up call to the global AIDS community. What we already suspected became the conference’s drumbeat: the ambitious 90-90-90 targets are unlikely to be met at the global level by 2020. There was hand-wringing. There were Rin-Tin-Tin jokes. There were journalistic “flourishes” that conflated challenges and/or got the facts wrong. But above the pessimistic din, the data that we should all be focused upon were beamed onto large-screen monitors from one end of the AIDS 2018 venue to another over 7 days. National and municipal programs presented on their 90-90-90 progress, without neglecting to mention challenges. And, the US President’s Emergency Plan for AIDS Relief program’s continued stream of public health impact analysis data spoke to movement from 90-90-90 toward HIV epidemic control in several countries.

The world is missing the evidence base by averaging the global response and looking at a single graphic—a graphic that hides the deeper truth. Some countries are making progress using the 90-90-90 approach to leverage their ability to find, reach, and link HIV-negative individuals to comprehensive HIV prevention programming. These country epidemics are slowing, and the expansion of new HIV infections is stabilized or dropping. Also hidden are the countries that are failing to make progress—look at the difference between the Russian Federation and the Ukraine. If you averaged those two countries together, the lack of progress in the Russian Federation overshadows the gains in the Ukraine. Controlling the global HIV epidemic is a community-by-community approach that will lead to country-by-country success.

Our experience in the HIV response has demonstrated that time-bound targets drive progress, promote accountability, and unite diverse stakeholders—motivating both behavior and investments. The “3 by 5” target, the world’s first ambitious HIV treatment target, called for placing 3 million people living with HIV in low- and middle-income countries on antiretroviral therapy (ART). The target was not met until 2009, but ART coverage tripled from 400 000 people to 1.3 million between 2003 and 2005, and funding for HIV programs increased by 60%.^1^ Well beyond what “3 by 5” achieved, the 90-90-90 targets served to reorient the global AIDS response to prioritize scale up of the most potent weapon in our armamentarium. As a result, some countries and municipalities around the world have met the targets, meaning that AIDS-related deaths have been curbed and new HIV infections averted. Botswana, Cambodia, Denmark, Iceland, Singapore, Sweden, and the United Kingdom have achieved 90-90-90, as have the Fast-Track Cities of Amsterdam, New York City, Melbourne, Paris, and San Francisco.^2^ However 90-90-90 is not a goal onto itself, but instead a key point on a trajectory toward getting to zero new HIV infections and zero AIDS-related deaths.

Still, a pessimistic streak permeated AIDS 2018. To be fair, it is all too easy to be pessimistic. Yes, the 90-90-90 targets may be missed in certain geographic regions and among key populations by 2020. But, I side with Winston Churchill in believing that: “The pessimist sees difficulty in every opportunity. The optimist sees the opportunity in every difficulty.” Framed within the context of our indisputable successes with HIV treatment as prevention, the power of the Undetectable=Untransmittable message,^3^ the PARTNER 2 confirmation that zero transmissions equal zero risk,^4^ and new evidence that on-demand pre-exposure prophylaxis (PrEP) is highly effective in men who have sex with men,^5,6^ our pace must quicken not wane. With no vaccine or cure available in short order (though we should continue to invest in their discovery), there is one viable option: speed up efforts to attain 90-90-90 everywhere and across all populations and age bands, including by eliminating structural and other barriers, while refining and expanding our approaches.

Talk of impending disaster with the emergence of global HIV drug resistance is not real.^7^ Such talk is not borne out by existing data, and moreover, it represents a distraction. Besides, the world is moving to dolutegravir (with due caution) to mitigate resistance. So, what is real? Talk of an HIV prevention crisis is real. We must recognize primary HIV prevention as the huge failure that it is and rethink and adequately fund a new approach that is designed by and for communities of affected individuals. Moreover, there exists an urgent need for scaled-up community engagement to find and test the missing millions of people who are unaware of their status. And, there is a human rights imperative to both facilitate universal access to viral load monitoring as a right for all people living with HIV and eliminate the unrelenting stigma which is a barrier to accessing and utilizing HIV services in every corner of the world.

The International AIDS Society—Lancet Commission reminds us that the HIV epidemic is not on track to end as a public health threat by 2030 and that it may rebound if corrective action is not soon taken.^8^ Even if the epidemic does not rebound, it is likely to remain a global challenge for the foreseeable future, which requires that we look at the promising successes achieved in country after country and see clearly what needs to be done to improve performance in underperforming countries. While 90-90-90 has contributed to many of these successes, there are opportunities to improve upon the approach. For example, HIV services should be de-siloed and integrated into general health care services, supported by strategic health financing and universal health coverage. Doing so successfully will require leveraging (rather than diminishing or ignoring) lessons learned from the gains that HIV programs have made through successive time-delimited campaigns with their measurable programmatic targets (eg, “3 by 5,” 90-90-90).

Any notion that missing the 2020 deadline for attaining 90-90-90 excuses throwing out a set of successful programmatic targets is wrong. We are agreed with “professional pessimists” that HIV treatment alone will not end the HIV epidemic. That is why a renewed commitment to the scaling up of HIV treatment services to attain the 90-90-90 targets by 2020 (or 2025) must be matched by a similar commitment to expanded access to primary HIV prevention within the context of a world that is liberated of HIV-related stigma and discrimination.
